# High Dynamic Range Turbo‐FLASH B1 Mapping for Characterizing Multi‐Transmit Multi‐Receive RF Arrays, Validated at 7 T and 11.7 T

**DOI:** 10.1002/mrm.70569

**Published:** 2026-09-17

**Authors:** Natalia Dudysheva, Franck Mauconduit, Michel Luong, Alexis Amadon, Nicolas Boulant, Vincent Gras

**Affiliations:** ^1^ Université Paris‐Saclay, CEA, CNRS, BAOBAB, NeuroSpin Gif sur Yvette France; ^2^ Université Paris‐Saclay, CEA, DRF, IRFU Gif sur Yvette France

**Keywords:** B1 mapping, parallel transmission, RF array, ultra‐high field MRI

## Abstract

**Purpose:**

Transmit (B_1_^+^) and receive (B_1_^−^) sensitivities of parallel‐transmit/receive RF array are a prerequisite for modern ultra‐high‐field MRI. Their accurate mapping remains challenging for coils with high transmit dynamic range, particularly in regions with low combined‐mode B_1_^+^ magnitude, where standard reconstruction is impaired by noise. This work presents a new B_1_^+^ and B_1_^−^ model‐based fitting approach for interferometric presaturated TurboFLASH (satTFL) data. With an optimized acquisition scheme, this method improves B_1_^+^/B_1_^−^ mapping accuracy.

**Methods:**

Voxel‐wise B_1_^+^ and B_1_^−^ are expressed as the product of a complex unit‐norm vector (phasor) and a scalar (efficiency). Phasors are estimated through singular value decomposition of satTFL signals, and efficiencies are recovered with fitting to the signal equation. This method was evaluated in phantom simulations for 7 T and 11.7 T 8Tx/32Rx RF arrays and compared with standard approaches. As it supports a variable number of pre‐saturated scans, different interferometric schemes were optimized for B_1_^+^ accuracy. Finally, schemes yielding acceptable B_1_^+^ error with minimal acquisition time were tested in vitro at 7 T and 11.7 T and in vivo at 7 T.

**Results:**

The proposed method provided lower median B_1_^+^ and B_1_^−^ errors than conventional satTFL methods and fewer outliers; the number of voxels with error > 30% is reduced from several dozens to zero. In vitro results were consistent with the simulations, and in vivo maps demonstrated good quality.

**Conclusion:**

The proposed method improves B_1_^+^ and B_1_^−^ mapping robustness to noise and enables more flexible satTFL acquisition scheme, allowing better trade‐offs between accuracy and scan time.

## 1 Introduction

The pursuit of higher SNR and improved spatial resolution in MRI has driven the transition to ultra‐high magnetic fields (UHFs) [1, 2] and development of dedicated multi‐transmit multi‐receive RF arrays [3, 4, 5]. These systems are indispensable for parallel transmission (pTx), which is increasingly important at UHFs [6, 7] for addressing pronounced wave effects and associated signal and contrast losses [1, 8]. However, effective image acquisition and reconstruction with such RF arrays require accurate characterization of the complex transmit (B_1_^+^ or Tx) and receive (B_1_^−^ or Rx) sensitivities of each array channel. In particular, accurate B_1_^+^ mapping is critical for computing optimal pTx dynamic RF shimming to improve excitation homogeneity [9, 10].

Among B_1_^+^ mapping techniques, presaturated turboFLASH (SatTFL) [11, 12] has been widely adopted, offering a good compromise between precision, acquisition speed, and SAR efficiency [13]. For multi‐transmit coils, a rapid SatTFL‐based approach: (1) acquires relative B_1_^+^ maps (I_rel_) for each of the N_TX_ transmit channels using a turboFLASH readout, (2) derives an absolute combined‐mode B_1_^+^ map from the ratio of the saturation‐prepared (I_sat_) to the non‐saturated (I_0_) turboFLASH images, and (3) scales I_rel_ using this map [14, 15]. However, accurate reconstruction requires SatTFL flip angles to remain within a limited dynamic range across the ROI, with the upper and lower bounds set by the validity of the assumed signal model and the noise floor, respectively [11, 13]. To reduce B_1_^+^ mapping errors, the interferometric approach was proposed [16, 17, 18]. Using N_TX_ independent linear channel combinations (LCCs), it acquires interferometric relative B_1_^+^ maps with higher homogeneity, and subsequently recovers channel‐specific B_1_^+^ maps by simple linear algebra.

A remaining challenge for noise‐robust B_1_^+^ reconstruction is the inhomogeneity of the combined mode (e.g., circularly polarized) itself. This aspect becomes apparent in abdominal 7 T MRI [19], neuroimaging at field strengths above 7 T [20], or for particular custom coil designs, such as those targeting extended regions [18, 21]. In such cases, even under the interferometric approach, noise propagation can be dramatic in regions with low combined‐mode B_1_^+^ because the ratios I_sat_/I_0_ and I_rel_/I_0_ are computed with I_0_ near the noise floor. Proposed solutions include acquisitions with varying driving voltages [22, 23], which are time‐consuming, or time‐interleaved acquisitions with two complementary transmit modes and adapted B_1_^+^ reconstruction algorithm [19].

In this work, we propose a new B_1_^+^ and B_1_^−^ reconstruction algorithm that relies on model‐based B_1_^+^ fitting of satTFL data from all receive channels and B_1_^+^‐encoding LCCs. Compared with previously presented methods [12, 18, 24, 25], this algorithm reduces noise‐induced errors in reconstructed B_1_ maps and offers greater flexibility in the choice of the LLCs for B_1_^+^ encoding. These advantages help minimize the adverse effect of a high transmit dynamic range of the combined mode. First, we formally describe the algorithm and demonstrate that the N_TX_‐dimensional complex B_1_^+^ can be estimated robustly despite the non‐convexity of the underlying least‐squares problem. Subsequently, a heuristic to optimize the LCCs is proposed. Our method is benchmarked using Monte Carlo simulations on simulated phantom B_1_^+^ maps and validated experimentally at 7 T and 11.7 T using two home‐made 8Tx32Rx head coils exempt of azimuthal symmetry. Finally, in vivo B_1_^+^ and B_1_^−^ maps at 7 T using 8Tx32Rx head coil are provided showing evidence of the method's practical applicability.

## 2 Theory

### 2.1 SatTFL Signal

As the B_1_ mapping methods treat every voxel of the acquired data independently, the derivations are presented for a single voxel without loss of generality. Let $N_{\mathrm{LCC}}$ ($N_{\mathrm{LCC}}>N_{\mathrm{TX}}$) be the total number of acquired satTFL images (pre‐saturated or not), and $N_{\mathrm{TX}}$ ($N_{\mathrm{RX}}$) the number of transmit (receive) channels. The complex transmit and receive sensitivities of the RF array are represented as the vectors $B_{1}^{+}\in {\mathbb{C}}^{N_{\mathrm{TX}}}$ (rad·s^−1^·V^−1^) and $B_{1}^{-}\in {\mathbb{C}}^{N_{\mathrm{RX}}}$ (a.u.), whereby each component ($B_{1,j}^{\pm }$) corresponds to one channel of the array. For the ith LCC ($1\leq i\leq N_{\mathrm{LCC}}$), we denote by $\alpha_{i}$ and $\beta_{i}$ the actual pre‐saturation and the readout flip angles (FA), respectively. The satTFL signal is then modeled as an $N_{\mathrm{LCC}}\times N_{\mathrm{RX}}$ complex matrix with elements:

(1) $$S_{i,j}=B_{1,j}^{-}\cos\left(\left|\alpha_{i}\right|\right)\sin\left(\beta_{i}\right)$$

For simplicity, we include scaling coefficients (e.g., proton density) in the receive sensitivities and neglect T_1_ relaxation assuming a sufficiently long repetition time of the saturation pulse (TR/T_1_> > 1) and small readout FAs [11, 12]. The LCCs underlying satTFL image formation are denoted by the unitless vectors $x_{i}$ and $y_{i}\in {\mathbb{C}}^{N_{\mathrm{TX}}}$ for the pre‐saturation and the readout pulses, respectively, each being unitary or zero (no excitation).

The LCC vectors are grouped into the so‐called presaturation and readout encoding matrices (EncMats) $X≔\left[x_{1}\;\cdots \;x_{N_{\mathrm{LCC}}}\right]\in {\mathbb{C}}^{N_{\mathrm{TX}}\times N_{\mathrm{LCC}}}$ and $Y≔\left[\begin{array}{lll} y_{1} & \cdots & y_{N_{\mathrm{LCC}}} \end{array}\right]\in {\mathbb{C}}^{N_{\mathrm{TX}}\times N_{\mathrm{LCC}}}$. With $\tau_{\mathrm{ref}}$ = 1 ms, $V_{\mathrm{ref}}$ the scanner's reference voltage, $\alpha_{\mathrm{nom}}$ and $\beta_{\mathrm{nom}}$ the nominal pre‐saturation and readout FAs, $I_{A}≔\frac{\alpha_{\mathrm{nom}}}{180}\cdot V_{\mathrm{ref}}\cdot \tau_{\mathrm{ref}}$ and $I_{B}≔\frac{\beta_{\mathrm{nom}}}{180}\cdot V_{\mathrm{ref}}\cdot \tau_{\mathrm{ref}}$ (V·s) the associated RF pulse integrals, the flip‐angle maps are given by $\alpha_{i}=I_{A}x_{i}^{T}B_{1}^{+}$ and $\beta_{i}=I_{B}y_{i}^{T}B_{1}^{+}$. With $s\left(B_{1}^{+}\right)$ denoting the transform:

(2) $$s:B_{1}^{+}\mapsto \left(\begin{array}{l} \cos\left(\left|\alpha_{1}\right|\right)\sin\left(\beta_{1}\right)\\ \vdots \\ \cos\left(\left|\alpha_{N_{\mathrm{LCC}}}\right|\right)\sin\left(\beta_{N_{\mathrm{LCC}}}\right) \end{array}\right)\in {\mathbb{C}}^{N_{\mathrm{LCC}}}$$

the signal model can be rewritten compactly as the product of column vector and row vector:

(3) $$S=s\left(B_{1}^{+}\right){B_{1}^{-}}^{T}\in {\mathbb{C}}^{N_{\mathrm{LCC}}\times N_{\mathrm{RX}}}$$

In what follows, satTFL acquisition consists of a so‐called pre‐sat bloc of $N_{\mathrm{SAT}}$ acquisitions with non‐zero α, followed by a no‐sat bloc consisting of $N_{\text{NoSAT}}=N_{\mathrm{LCC}}-N_{\mathrm{SAT}}$ images with α=0. We thus rewrite presaturation and readout encoding matrices as:

(4a) $$X:\;=\left[\begin{array}{ll} X^{\left(\mathrm{sat}\right)} & 0 \end{array}\right]$$

(4b) $$Y:\;=\left[\begin{array}{ll} Y^{\left(\mathrm{sat}\right)} & Y^{\left(0\right)} \end{array}\right]$$

### 2.2 Standard B_1_ ^+^ Reconstruction

The conventional reconstruction methods used for comparison with the proposed approach are described in detail in the Supporting Information. The standard hybrid [14, 15, 26] satTFL protocol (**HybM**) requires $N_{\mathrm{LCC}}=N_{\mathrm{TX}}+2$ SatTFL acquisitions. With c being the combined mode of the Tx array and M the interferometric matrix (e.g., all‐channels‐on‐one‐inverted [17]), the B_1_^+^ encoding scheme of HybM reads: $X=\left[\begin{array}{lll} c & 0 & 0 \end{array}\right]$ and $Y=\left[\begin{array}{lll} c & c & M \end{array}\right]$. The pairwise [12, 25] method (**PwM**) uses $N_{\mathrm{LCC}}=2N_{\mathrm{TX}}+1$ images and therefore introduces some redundancy to improve robustness. Its B_1_^+^ encoding scheme is $X=\left[\begin{array}{lll} M & 0 & 0 \end{array}\right]$ and $Y=\left[\begin{array}{lll} C & M & c \end{array}\right]$, where $C=\left[\begin{array}{lll} c & \cdots & c \end{array}\right]$ matches the dimensions of M.

### 2.3 Proposed FitM Algorithm

The entry point of the algorithm is the TFL data matrix $D\in {\mathbb{C}}^{N_{\mathrm{LCC}}\times N_{\mathrm{RX}}}$ with each column corresponding to the signal collected by one receive channel, and each row to one B_1_^+^‐encoding LCC. The measured signal is matched to the theoretical model S (Equation 3). To find the transmit and receive sensitivities, we rewrite them as

(5) $$B_{1}^{\pm }=\sigma^{\pm }b_{1}^{\pm }$$

with $\sigma^{\pm }≔{\left\|B_{1}^{\pm }\right\|}_{2}$. Here, the vectors $b_{1}^{\pm }$ encode channel‐specific complex information and have unit norm; they are called the transmit/receive phasors, respectively. The scalars $\sigma^{\pm }$ represent the sum‐of‐squares (SoS) transmit/receive profiles across all channels. These components are estimated separately as follows (see also Figure 1).

**FIGURE 1:**
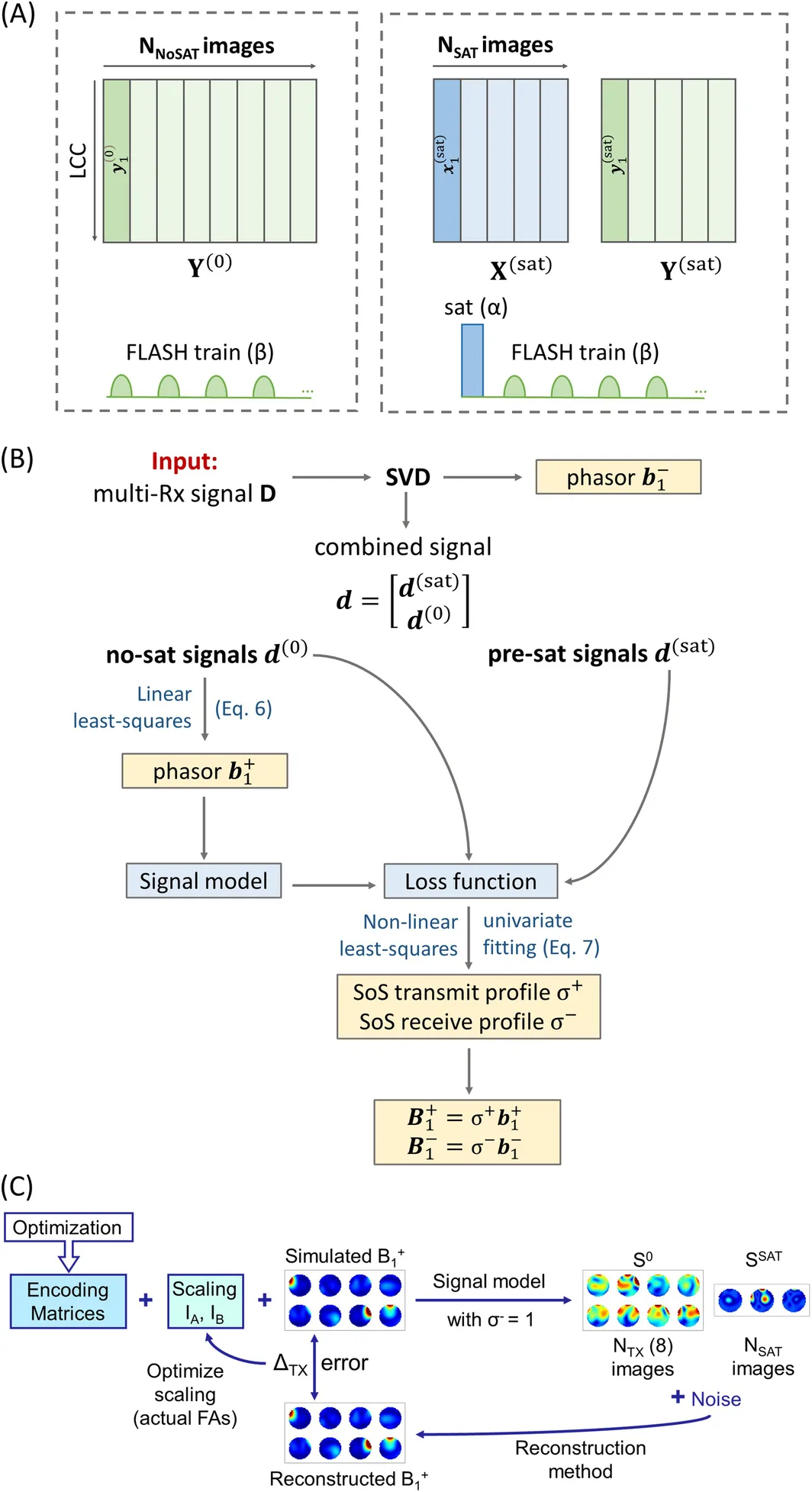
(A) Top: Structure of the LCC encoding matrices for the pre‐saturation and readout pulses. Each column corresponds to the LCC applied in a given acquisition, and the total number of columns equals the number of acquisitions. Bottom: Illustration of the two sequence blocks (no‐sat and pre‐sat acquisitions). (B) Schematic overview of the proposed B_1_^+^ reconstruction. (C) Simulation‐based framework for assessing error propagation in B_1_^+^ reconstruction and adjusting the RF pulse integrals I_A_ and I_B_ to minimize these errors.

Step 1: We project the acquired noisy data D to a rank‐one matrix consistent with the theoretical model S [27, 28]. Applying the singular value decomposition (SVD) to D and retaining only the first principal component yields the best rank‐one approximation $d{\left(\hat{b_{1}^{-}}\right)}^{T}$, where $d\in {\mathbb{C}}^{N_{\mathrm{LCC}}}$ and $\hat{b_{1}^{-}}\in {\mathbb{C}}^{N_{\mathrm{RX}}}$ are column vectors and $\hat{b_{1}^{-}}$ is unit‐norm. Under the signal model, $\hat{b_{1}^{-}}$ provides an estimate of the receive phasor, while d corresponds to the combined signal $\sigma^{-}s\left(B_{1}^{+}\right)$. Similarly to Equation (4), we decompose signal the d into pre‐sat and no‐sat blocs, that is, $d=\left[\begin{array}{l} d^{\left(\mathrm{sat}\right)}\\ d^{\left(0\right)} \end{array}\right]$.

Step 2: The transmit phasor is estimated using the no‐sat data $d^{\left(0\right)}$. By linearizing sin(β) for small β, the problem reduces to a linear least‐squares formulation with a unique solution:

(6) $$\hat{b_{1}^{+}}=\frac{{\left({Y^{\left(0\right)}}^{T}\right)}^{†}d^{\left(0\right)}}{{\left\|{\left({Y^{\left(0\right)}}^{T}\right)}^{†}d^{\left(0\right)}\right\|}_{2}}$$

where † denotes the Moore–Penrose pseudo‐inverse operator.

Step 3: The transmit phasor estimate $\hat{b_{1}^{+}}$ is substituted into the analytical signal model, and the remaining unknowns, $\sigma^{\pm },$ are recovered by model‐based fitting to the signal d:

(7) $$\left(\hat{\sigma^{+}},\hat{\sigma^{-}}\right)=\underset{\sigma^{+},\sigma^{-}}{\text{argmin}}\left({\left\|d-\sigma^{-}s\left(\sigma^{+}\hat{b_{1}^{+}}\right)\right\|}_{2}\right)$$

As detailed in Section S2, this problem can be reduced to a one‐dimensional nonlinear least‐squares optimization in $\sigma^{+}$, while $\hat{\sigma^{-}}$ is obtained analytically via linear least‐squares.

Step 4: The entire transmit and receive sensitivities are reconstructed as $\hat{\sigma^{\pm }}\hat{b_{1}^{\pm }}$.

### 2.4 satTFL Acquisition Scheme Optimization

In what follows, we propose a method to optimize EncMats, which significantly affect B_1_ reconstruction quality. The proposed procedure relies on a reference B_1_^+^ distribution obtained from highly averaged measurements or electromagnetic simulations, and the following heuristic. We denote by $B_{1,\mathrm{ref}}^{+}$ the $N_{\mathrm{TX}}\times N_{v}$ matrix of reference transmit sensitivities, whose columns correspond to transmit channels and rows to voxels of interest. For a given LCC z we define its FA pattern as row vector:

(8) $$p_{z}≔\frac{\left|z^{T}B_{1,\mathrm{ref}}^{+}\right|}{\left\langle \left|z^{T}B_{1,\mathrm{ref}}^{+}\right|\right\rangle }\in {\mathbb{R}}^{1\times N_{v}}$$

where ⟨·⟩ denotes averaging across $N_{v}$ voxel. By extension, for an encoding matrix $Z:\;=\left[z_{1}\cdots z_{n}\right]\in {\mathbb{R}}^{N_{\mathrm{TX}}\times n}$ of n LCCs, we define the corresponding FA‐pattern matrix as:

(9) $$P_{Z}≔\left[\begin{array}{l} p_{z_{1}}\\ \vdots \\ p_{z_{n}} \end{array}\right]\in {\mathbb{R}}^{n\times N_{v}}$$

The FitM approach accommodates encoding schemes of the general form ((4a), (4b)). To limit the design space, we choose:

1. A square matrix $Y^{\left(0\right)}$, that is, $N_{\text{NoSAT}}=N_{\mathrm{TX}}$.
2. A number of pre‐sat scans in the range $1\leq N_{\mathrm{SAT}}\leq N_{\mathrm{TX}}$.
3. Identical LCCs for the saturation and readout pulses in the pre‐sat scans, that is, $X^{\left(\mathrm{sat}\right)}=Y^{\left(\mathrm{sat}\right)}.$

We constraint the maximum condition number of $Y^{\left(0\right)}$ and $Y^{\left(\mathrm{sat}\right)}$ to 2 to ensure linear independence of the LCCs. A maximum condition number of 2 appears as a compromise between the orthogonal‐matrix case (cond = 1) and the “all‐channels‐on‐one‐inverted” encoding matrix [17] (cond = 3). To compute $Y^{\left(0\right)}$ and $Y^{\left(\mathrm{sat}\right)}$, we promote minimal spatial variation of the respective FA patterns $P_{Y^{\left(0\right)}}$ and $P_{Y^{\left(\mathrm{sat}\right)}}$ by solving:

(10) $$\underset{Z}{\text{argmin}}\left({\left\|P_{Z}-1\right\|}_{\mathrm{Fro}}\right)s.t.\;\text{cond}\left(Z\right)\leq 2$$

where ${\left\|\cdot \right\|}_{\mathrm{Fro}}$ is the Fröbenius norm, and Z has dimensions $N_{\mathrm{TX}}\times N_{\mathrm{TX}}$ and $N_{\mathrm{TX}}\times N_{\mathrm{SAT}}$ when optimizing $Y^{\left(0\right)}$ and $Y^{\left(\mathrm{sat}\right)}$, respectively.

With appropriate choice of the RF integrals I_A_ and I_B_, the reference FA profiles are brought within the method's stability range. The parameters I_A_ and I_B_ are optimized using Monte Carlo simulations following a procedure sketched in Figure 1. There, the B_1_^+^ reconstruction algorithm is applied to synthetic satTFL signals with additive white Gaussian noise, and the resulting estimate $\hat{B_{1}^{+}}$ is compared with the ground truth $B_{1}^{+}$ using the error metric:

(11) $$\Delta_{\mathrm{TX}}\left(r\right)=\frac{\left\|B_{1}^{+}\left(r\right)-\hat{B_{1}^{+}}\left(r\right)\right\|}{\left\|B_{1}^{+}\left(r\right)\right\|}$$

The I_A_ and I_B_ values, minimizing the average error $\left\langle \Delta_{\mathrm{TX}}\right\rangle$ across voxels and Monte Carlo repetitions are finally selected. Additional constraints are imposed to keep *α* ≤ 180° and *β* ≤ 30° to avoid deviations from satTFL signal model (Equation 1).

## 3 Methods

### 3.1 RF Arrays

We studied the proposed approach at 7 T and 11.7 T using in‐house developed 8Tx32Rx arrays with the same size and architecture, referred hereinafter as to Avanti2 and Iseult, respectively [29, 30]. Electromagnetic simulations for both coils were performed using finite element method solver Ansys HFSS (Ansys, PA, USA) for a 16 cm‐diameter spherical agar phantom (ε_r_ = 72, *σ* = 0.8 S/m) and a head model of a 26‐year‐old female (“Virtual Family” v2.0.1 [31]). The transmit field maps were exported with a (5 mm)^3^ resolution; the receive profile was not considered, assuming $N_{\mathrm{RX}}=1$ and $B_{1}^{-}=1$. The simulations were used to optimize EncMats and benchmark B_1_^+^ reconstruction methods.

### 3.2 Method Benchmarking

Numerical optimizations were performed in Matlab (R2022b) using the SQP algorithm implemented in fmincon. First, we designed EncMats and evaluated HybM, PwM, and FitM using spherical phantom simulations. For HybM and PwM, the acquisition schemes (Section) used the interferometric matrix **M** optimized with the proposed heuristic and the combined mode c with max‐to‐min transmit‐profile ratios of 112 for Avanti2 and 116 for Iseult. For FitM, we considered optimized EncMats (Section 2.4) with N_sat_ = 1…8 pre‐saturated acquisitions. RF pulse integrals were optimized using 10 Monte Carlo simulations for each (I_A_, I_B_) pair. SatTFL signals were synthesized with complex Gaussian noise with a standard deviation of 10^−3^ (SNR of 50–100 in non‐saturated images). Using the corresponding acquisition schemes and optimal (I_A_, I_B_), each reconstruction method was evaluated with 100 Monte Carlo repetitions to assess noise robustness. One dataset reconstruction took ˜20 s on a workstation with 8‐core Intel Core i9‐9880H CPU. Alongside the transmit‐profile error $\Delta_{\mathrm{TX}}$ (Equation 11), we quantified the deviations of the estimated reception profile from the ground‐truth value as $\Delta_{\mathrm{RX}}≔\left\|\hat{\sigma^{-}}-1\right\|$. To evaluate the sensitivity of EncMat optimization to a particular choice of reference B_1_^+^ distribution, we assessed FitM performance using phantom‐derived EncMats in head‐model simulations, which are expected to better reflect actual head scans.

### 3.3 Experimental Validation

The acquisition schemes with the shortest scan time (minimum $N_{\mathrm{SAT}}$) and negligible number of local mapping errors exceeding 30% (0–3 voxels) in both phantom and brain simulations were selected for in vivo applications. For the Avanti2 coil, satTFL acquisitions were performed on a Magnetom 7 T MRI scanner (Siemens Healthineers, Erlangen, Germany). After experimental validation on a spherical phantom, in vivo B_1_^+^/B_1_^−^ mapping was performed using FitM with N_sat_ = 3 and I_a_ reduced by 10% to prevent potential *α* > 180°. The in vivo experiments were approved by the local ethics committee and conducted on six healthy volunteers, who gave written informed consent. For the Iseult coil, in vitro tests were performed on the 11.7 T Iseult MR system (Siemens Healthcare, Erlangen, Germany), using a spherical phantom.

We used custom‐developed SatTFL sequences with a minimum‐phase Shinnar‐Le Roux (SLR) pulse for magnetization preparation [32]. At 7 T, TR was 10 s for the no‐sat acquisitions and 20 s for the pre‐sat acquisitions and TE = 1.7 ms. At 11.7 T, both TRs were 15 s, and TE = 1.89 ms. In both cases, the in‐plane resolution was (5 mm)^2^, the readout slice thickness was 2.5 mm, while the saturated slice thickness was 5 mm [33], and 36 axial slices were acquired in an interleaved manner.

## 4 Results

Figure 2 and Table S2 summarize the B_1_^+^ and B_1_^−^ error metrics obtained from spherical phantom simulations: the median Med(Δ), the 99th percentile $P_{99}\left(\Delta \right)$, and the fraction of voxels with error exceeding 30% $D_{30}\left(\Delta \right)$. The corresponding acquisition times, measurement numbers, and average actual FAs for each acquisition scheme are also reported in the Supporting Information.

**FIGURE 2:**
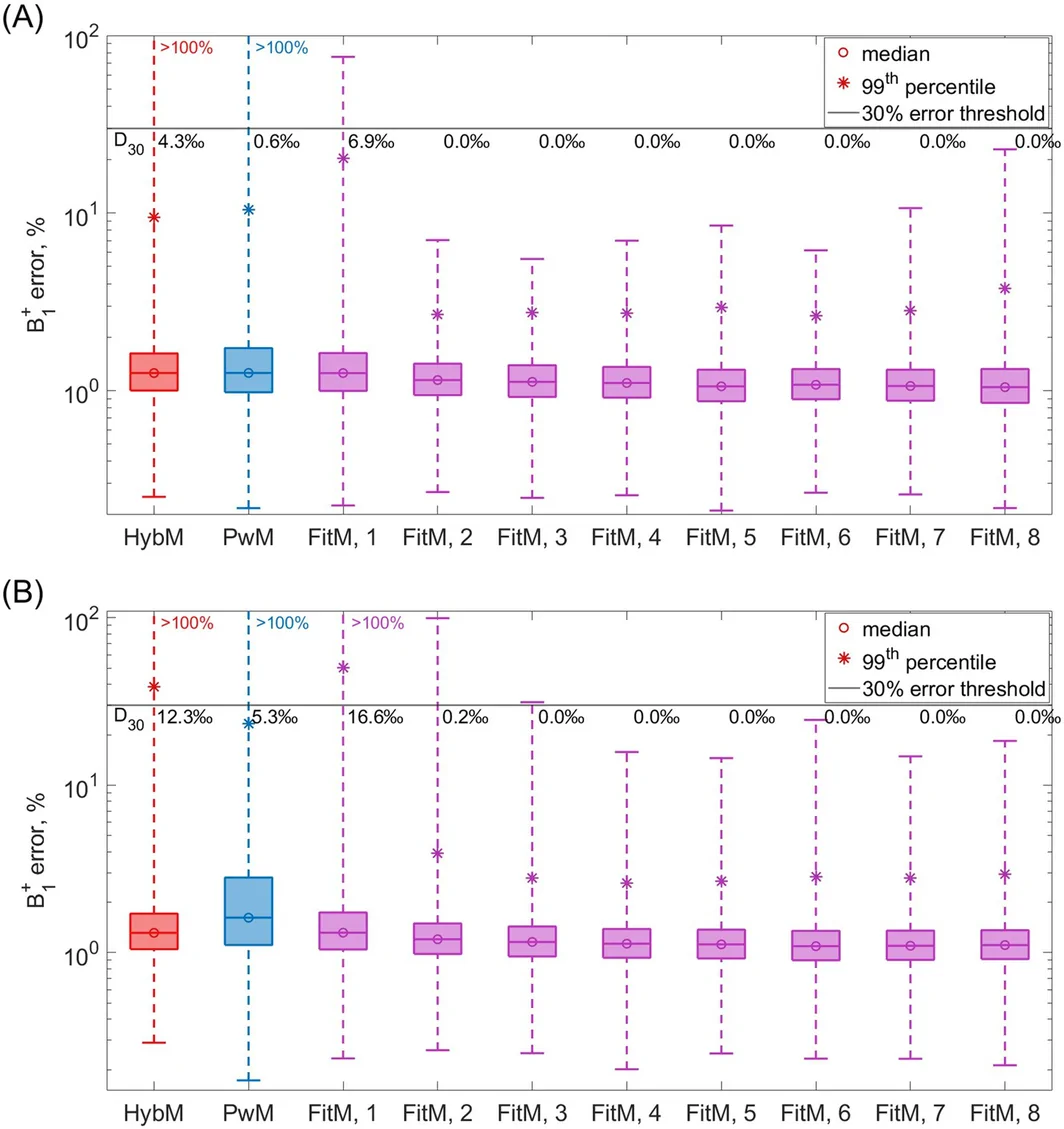
B_1_^+^ error distributions (log scale) for the 7 T Avanti2 coil (A) and the 11.7 T Iseult coil (B) obtained in phantom simulations. Boxplots are shown for the HybM (red), PwM (blue), and FitM (violet; N_sat_ = 1–8) methods applied to simulated data with added Gaussian noise (SNR = 1000; 100 repetitions). The whiskers extend from the minimum to the maximum values, and the median and the 99th percentile are indicated. The fraction of voxels with errors exceeding 30% (D_30_) is reported next to each boxplot in per mille (‰).

For the conventional methods, the number of “problematic” voxels with $\Delta_{\mathrm{TX}}>30\%$ appears substantial: HybM provides $D_{30}\left(\Delta_{\mathrm{TX}}\right)$ of 0.43% (about 63 voxels) for Avanti2 and 1.23% (∼193 voxels) for Iseult; PwM yields 0.06% (9 voxels) for Avanti2 and 0.53% (83 voxels) for Iseult. In contrast, FitM reduces $D_{30}\left(\Delta_{\mathrm{TX}}\right)$ to zero at N_sat_ ≥ 2 for Avanti2 and N_sat_ ≥ 3 for Iseult; moreover, it yields lower median B_1_^+^ error (< 1.32%). However, using only one saturation cycle is prone to outliers with $P_{99}\left(\Delta_{\mathrm{TX}}\right)$ reaching 20.4% for Avanti2 and 50.4% for Iseult. At least two complementary saturation modes are required to stabilize the method. Increasing N_sat_ up to 6 generally reduces the reconstruction errors; however, beyond six pre‐saturated acquisitions, B_1_^+^ and B_1_^−^ errors increase slightly (see Table S2). This unexpected result may reflect the difficulty of constructing a large number of complementary LCCs while maintaining FAs within the optimal range under the proposed EncMat optimization strategy. This limitation could potentially be overcome with an alternative EncMat optimization method.

Figure 3 and Table S3 report the B_1_^+^ reconstruction accuracy of FitM with phantom‐optimized EncMats (N_sat_ = 1…8) evaluated in head‐model simulations. Somewhat expected, the change in B_1_^+^ field distribution between the sphere and the head model, owing to differences in geometry and dielectric properties, slightly increased the reconstruction error. Yet, $D_{30}\left(\Delta_{\mathrm{TX}}\right)$ remained below 0.01% (< 2 aberrant voxels) and $\mathrm{Med}\left(\Delta_{\mathrm{TX}}\right)$ below 1.2% for the Avanti2 coil provided N_sat_ > 2, and for the Iseult coil provided N_sat_ > 4. For subsequent experiments, we used N_sat_ of 3 and 5 for Avanti2 and Iseult, respectively.

**FIGURE 3:**
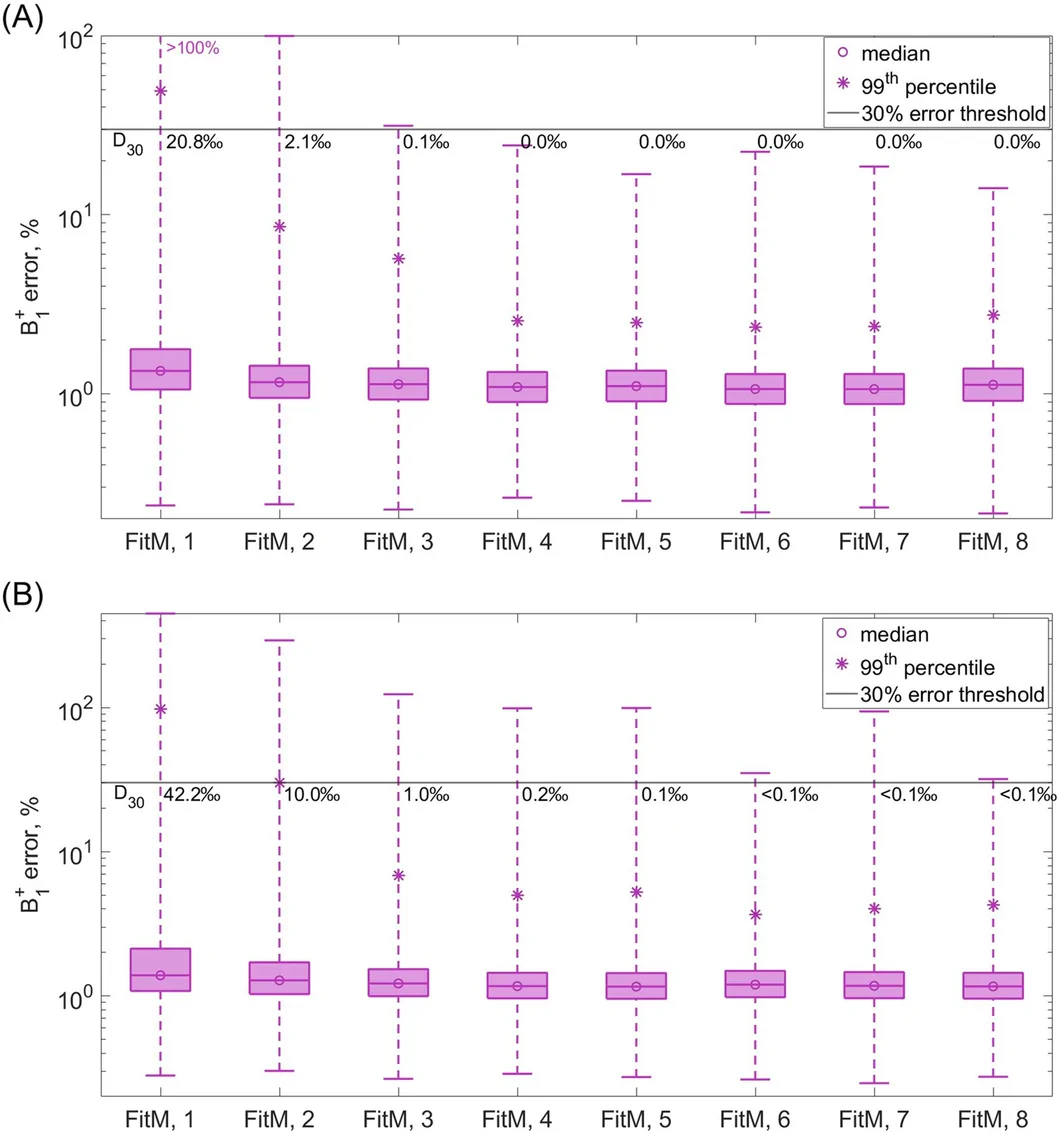
B_1_^+^ error distributions (log scale) for the 7 T Avanti2 coil (A) and the 11.7 T Iseult coil (B) obtained in head model simulations. Boxplots are shown for the FitM method with N_sat_ = 1–8 applied to simulated data with added Gaussian noise (SNR = 1000; 100 repetitions). The whiskers extend from the minimum to the maximum values, and the median and the 99th percentile are indicated. The fraction of voxels with errors exceeding 30% (D_30_) is reported next to each boxplot in per mille (‰).

Figure 4 shows the transmit (*σ* ^+^) and receive (*σ* ^−^) SoS profiles reconstructed from synthetic noisy data (spherical phantom simulation) using HybM, PwM, and FitM for both coils; experimental results for FitM are also provided. The HybM method (TA: 2 min) results in highly noise‐affected voxels in both transmission and reception maps. The PwM method (TA: 4 min 20 s) improves reconstruction performance at the cost of a longer acquisition time but still exhibits non‐negligible discontinuities in the reconstructed fields. The FitM method with the proposed acquisition schemes (N_sat_ = 3, TA: 2 min 30 s for Avanti2 and N_sat_ = 5, TA: 3 min 15 s for Iseult) returns the best reconstructions at both 7 T and 11.7 T, producing smooth B_1_^+^ distributions without outliers.

**FIGURE 4:**
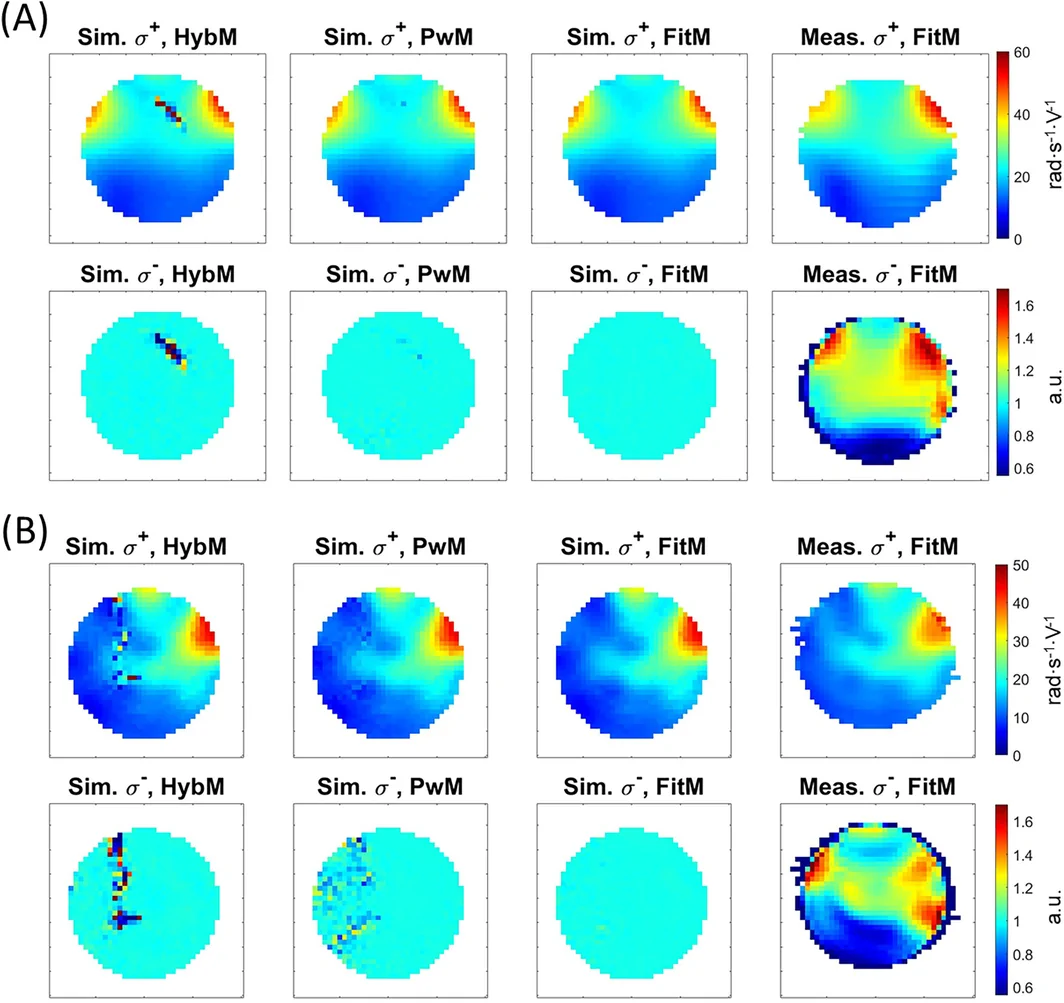
Reconstructed SoS transmit *σ* ^+^ (top row) and receive *σ* ^−^ (bottom row) profiles for a spherical phantom using the (A) 7 T Avanti2 coil and (B) 11.7 Iseult coil. The central sagittal slice is shown. From left to right: HybM, PwM, and FitM with N_SAT_ = 3 (A) or N_SAT_ = 5 (B), evaluated in simulations; the last column shows the experimental results obtained with FitM. For the experimental data, *σ* ^−^ is normalized to its mean over the ROI.

Figure 5 shows the in vivo 7 T B_1_^+^ maps acquired with the Avanti2 using the N_sat_ = 3 scheme and reconstructed with FitM. The SoS transmit and receive profiles $\sigma^{\pm }$ exhibit no visible discontinuities, suggesting the absence of major reconstruction errors. The reconstructed receive profiles for all Rx channels are provided in Figure S1.

**FIGURE 5:**
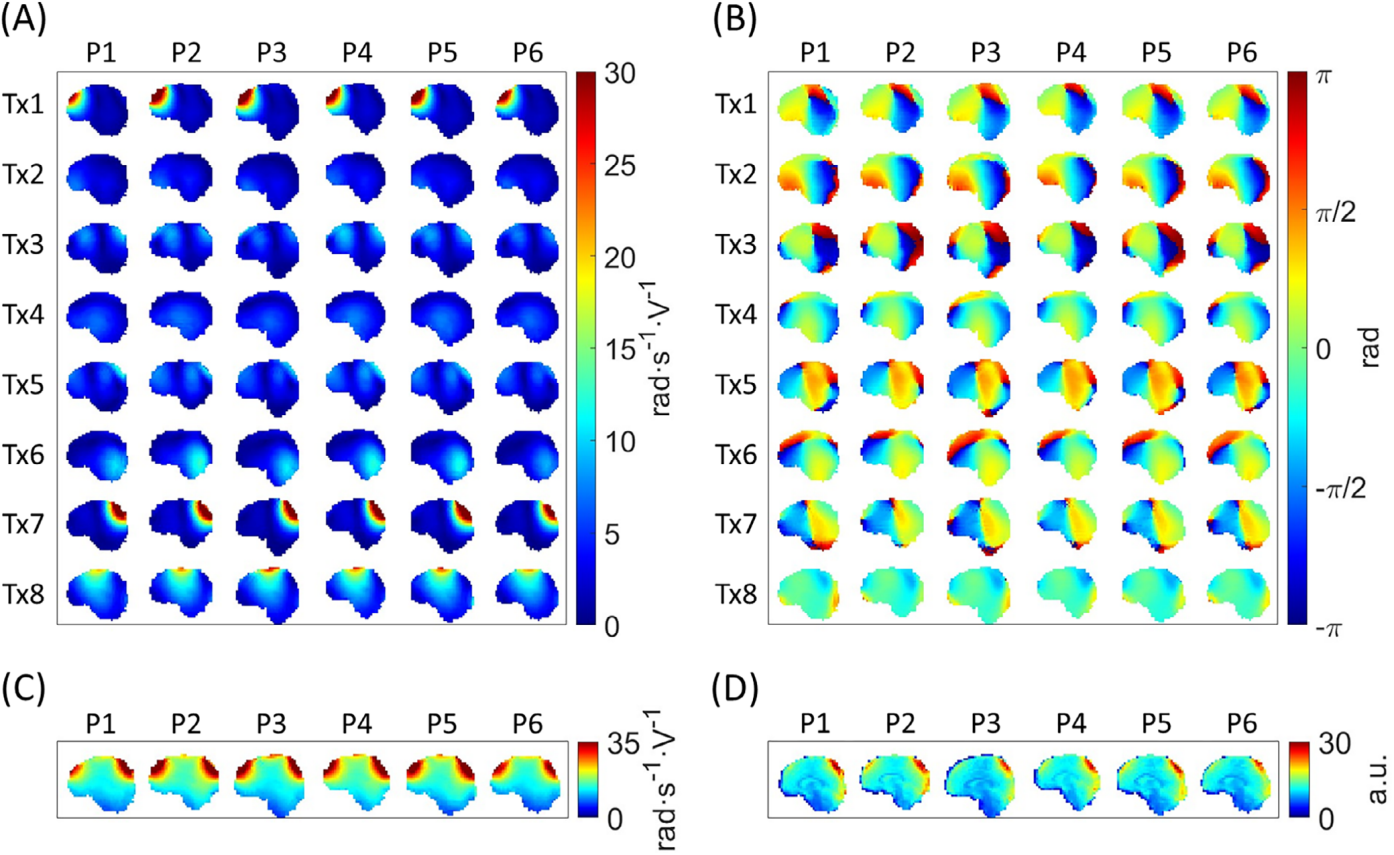
7 T in vivo data acquired in six participants (P1‐P6) with the Avanti2 coil using N_SAT_ = 3 acquisition scheme (TA: 2 min 30 s) and reconstructed with the FitM approach. (A) Magnitude and (B) phase of the B_1_^+^ maps for each transmit channel (Tx1‐Tx8). (C) Sum‐of‐squares transmit profile (*σ* ^+^). (D) Sum‐of‐squares receive profile (*σ* ^−^).

## 5 Discussion and Conclusions

This work presents a new model‐based fitting approach on interferometric satTFL data, enabling accurate transmit and receive sensitivity mapping. Its utility was demonstrated for two parallel‐transmit RF coils designed for head imaging at 7 T and 11.7 T, exhibiting large transmit dynamic range in the combined mode. At a modest increase in acquisition time relative to the conventional hybrid method (20s at 7 T and 45 s at 11.7 T), our approach reduced the $D_{30}$ metric to negligible levels, whereas the standard method yielded values of up to 4% (several dozen outlier voxels). Additionally, FitM provided generally lower median error than conventional approaches, potentially enhancing mapping quality under limited SNR. These improvements benefit the stability and accuracy of quality assurance routines, quantitative data correction, or pTx pulse design at UHF. In addition, the proposed method provides accurate receive sensitivity maps, useful for SENSE reconstruction [34], SNR‐optimal image combination [35], and quantitative SNR assessment. The results experimentally obtained on phantoms were consistent with simulation predictions. However, at 11.7 T, greater fitting error and instability were observed at small N_sat_, likely due to experimental conditions and unaccounted effects (e.g., T_1_ recovery, slice profile errors, off‐resonances). The 7 T in vivo maps obtained with the proposed approach showed good quality.

FitM provides flexibility in EncMat design and the number of pre‐saturated acquisitions, allowing a trade‐off between acquisition time and reconstruction accuracy. The acquisition scheme presented here was constructed heuristically using a simple cost function to generate LCCs with desirable properties, although alternative optimization strategies could be explored. Specifically, for pre‐saturation and readout, we promoted well‐conditioned EncMats and a minimal dynamic range of the resulting FA profiles to maintain FAs within an optimal range across voxels. Notably, α>20° in at least one pre‐saturated acquisition is required to robustly identify the local minimum of the objective function in (7), whereas α≤180° is imposed by the periodicity of the cosine function.

Moreover, our signal model assumed sin*β*≈*β* for transmit phasor estimation and neglected T_1_ relaxation, which is valid at low readout FAs. Although LCCs were optimized to limit flip‐angle variation, substantial dispersion remained. While the mean readout FA was below 10° (see ⟨*β*⟩ in the Supporting Information Tables), local values reached up to 30° for some LCCs, potentially introducing non‐negligible T_1_ effects and deviations from the assumed linear regime [24, 36]. Potential bias could be mitigated by reducing the readout RF pulse integral I_B_ at some SNR expense. T_1_ relaxation can also be incorporated into the ground‐truth signal when optimizing (I_A_, I_B_). In practice, when the ground truth is unknown, model bias may be assessed from the fitting residual or calibration measurements acquired at different driving voltage levels [23]. We also neglected off‐resonance effects and assumed ideal slice profile. If found to bias the results, both can be incorporated into the signal model [33].

Using a rough reference B_1_^+^ distribution for EncMat optimization provided satisfactory in vivo results due to the smooth spatial variation of B_1_^+^. However, we recommend slightly decreasing the RF pulse integrals I_a_ and I_b_ (e.g., by 10%) for the in vivo acquisitions to prevent risk that the saturation/readout FAs exceed 180°/30°. EncMat optimization could naturally be performed on a more representative reference B_1_^+^ distribution, or even actual B_1_^+^ maps. It could further be refined iteratively as the reference B_1_^+^ maps become progressively more accurate and representative. With appropriate adaptation of the encoding schemes, the FitM can be extended to any coil designs, anatomical regions, and field strengths. Its primary advantage is its ability to cope with pTx MRI setups with a large combined‐mode dynamic range, which facilitates the testing and use of custom RF arrays. Such setups are recurrent at ultra‐high field strengths beyond 10 T in the head and beyond 3 T in the body.

## Funding

This work was supported by the Agence Nationale de la Recherche (ANR) under Grant ANR‐21‐CE19‐0028 (MOSAR), France 2030 Future Investment Program, Grant ANR‐21‐ESRE‐0006, ESR/EquipEx+, PRESENCE Project.

## Conflicts of Interest

The authors declare no conflicts of interest.

## Supporting information


**Table S1:** Notation used.
**Table S2:** FitM evaluation for the 7 T Avanti2 and 11.7 T Iseult coils using spherical phantom simulations.
**Table S3:** FitM evaluation for the 7 T Avanti2 and 11.7 T Iseult coils using head model simulations.
**Figure S1:** In vivo B_1_^−^ maps, 7 T Avanti2 coil.

## Data Availability

The data that support the findings of this study are openly available in the B1RecoPTX GitHub repository (release v1.0.0) at https://github.com/VincentGras/B1RecoPTX/releases/tag/v1.0.0.
