# Supplementary material for: High Dynamic Range Turbo‐FLASH B1 Mapping for Characterizing Multi‐Transmit Multi‐Receive RF Arrays, Validated at 7 T and 11.7 T
Source: Magn Reson Med. 2026 Sep 17;97(1):240–9. doi: 10.1002/mrm.70569 (PMC13746065; doi:10.1002/mrm.70569)
Supplement: Supplementary file 1 [file MRM-97-240-s001.docx]

# **Supplementary Materials**

## **S1. Standard satTFL-based reconstruction of B_1_^+^ maps**

The *hybrid* B_1_^+^ reconstruction method (HybM) comprises $N_{\mathrm{TX}}+2$ acquisition cycles. The saturation (**X**) and readout (**Y**) encoding matrices are given by:

$\mathbf{X}=\left[ \begin{matrix} \boldsymbol{c} & \boldsymbol{0} & \boldsymbol{0} \end{matrix} \right]$	[S1a]

$\mathbf{Y}=\left[ \begin{matrix} \boldsymbol{c} & \boldsymbol{c} & \mathbf{M} \end{matrix} \right]$	[S1b]

Here, $\boldsymbol{c}\in\mathbb{C}^{N_{\mathrm{TX}}}$ is the combined-mode vector and $\mathbf{M}\in\mathbb{C}^{N_{\mathrm{TX}}\times N_{\mathrm{TX}}}$ is the interferometric matrix (e.g., all-channels-on-one-inverted). The reconstruction is based on a linear approximation of the signal with respect to β, assuming that β remains sufficiently small (for simplicity, the signal combined across receive channels is considered):

$S_{i} =\sigma^{-}\beta_{i}\cos\left( \left| \alpha_{i} \right| \right).$	[S2]

The index i identifies the corresponding SatTFL acquisition. HybM estimates $\boldsymbol{B}_{1}^{+}$ from the experimentally measured data, involving two divisions by the reference combined-mode signal to retrieve the FA maps of the individual LCCs. Denoting the experimentally measured signals as $\boldsymbol{d}\boldsymbol{:}=\left[ \begin{matrix} d_{1}^{(sat)} & d_{2}^{(0)} & d_{3}^{(0)} & \cdots& d_{N_{\mathrm{TX}}+2}^{(0)} \end{matrix} \right]$, where the superscripts (sat) and (0) denote acquisitions with and without pre-saturation, respectively, the reconstruction procedure can be described by the following steps:

1. $\left| \alpha_{1} \right|:= \arccos\left( \mathfrak{R}\left( \frac{d_{1}^{(sat)}}{d_{2}^{(0)}} \right) \right)$;
2. $\boldsymbol{\beta}_{\mathrm{interf}}\boldsymbol{:}\boldsymbol{=}\left[ \beta_{3}\ldots\beta_{N_{\mathrm{TX}}+2} \right]^{T}= \frac{I_{B}}{I_{A}}\left| \alpha_{1} \right|\frac{\left[ d_{3}^{(0)}\boldsymbol{\ldots}d_{N_{\mathrm{TX}}+2}^{(0)} \right]^{T}}{d_{2}^{(0)}}$;
3. $\hat{\boldsymbol{B}_{1}^{+}}=\frac{1}{I_{B}}\left( \mathbf{M}^{T} \right)^{-1}\boldsymbol{\beta}_{\mathrm{interf}}$

For voxels where the combined-mode flip angle is low, $d_{2}^{(0)}$ approaches the noise level. Its presence twice in the denominator amplifies noise and results in outliers in the reconstructed B_1_^+^ maps. On the other hand, increasing the readout FA to gain SNR introduces regions where the assumed linearity in β breaks down and can result in stronger T_1_-weighting effect.

The alternative *pairwise* method (PwM) includes ${2N}_{\mathrm{TX}}+1$ acquisition cycles in total, of which $N_{\mathrm{TX}}$ are acquired with pre-saturation. The associated saturation and readout encoding matrices are given by:

$\mathbf{X}=\left[ \begin{matrix} \mathbf{M} & \boldsymbol{0} & \boldsymbol{0} \end{matrix} \right]$	[S3a]

$\mathbf{Y}=\left[ \begin{matrix} \mathbf{C} & \mathbf{M} & \boldsymbol{c} \end{matrix} \right]$	[S3b]

with $\mathbf{C}$ formed by repeating $\boldsymbol{c}$ as columns to match the dimensions of the $\mathbf{M}$.

With corresponding acquired signals $\boldsymbol{d}\boldsymbol{:}=\left[ \begin{matrix} d_{1}^{(sat)} & \ldots& d_{N_{\mathrm{TX}}}^{(sat)} & d_{N_{\mathrm{TX}}+1}^{(0)} & \ldots& d_{2N_{\mathrm{TX}}}^{(0)} & d_{2N_{\mathrm{TX}}+1}^{(0)} \end{matrix} \right]$, the reconstruction algorithm is written as:

1. $\left| \boldsymbol{\alpha}_{\mathrm{interf}} \right|:=\left[ \alpha_{1}\ldots\alpha_{N_{\mathrm{TX}}} \right]^{T}=acos\left( \mathcal{R}\left( \frac{\left[ d_{1}^{(sat)}\boldsymbol{\ldots}d_{N_{\mathrm{TX}}}^{(sat)} \right]^{T}}{d_{2N_{\mathrm{TX}}+1}^{(0)}} \right) \right)$ ,

2. $\mathrm{angle}\left( \boldsymbol{\alpha}_{\mathrm{interf}} \right)=\mathrm{angle}\left( \left[ \beta_{N_{\mathrm{TX}}+1}\ldots\beta_{2N_{\mathrm{TX}}} \right]^{T} \right)=angle\left( \left( d_{2N_{\mathrm{TX}}+1}^{(0)} \right)^{*}\left[ d_{N_{\mathrm{TX}}+1}^{(0)}\boldsymbol{\ldots}d_{{2N}_{\mathrm{TX}}}^{(0)} \right]^{T} \right)$

3. $\boldsymbol{\alpha}_{\mathrm{interf}}\boldsymbol{=}\left| \boldsymbol{\alpha}_{\mathrm{interf}} \right|\boldsymbol{\cdot}\exp\boldsymbol{(}i\cdot angle\left( \boldsymbol{\alpha}_{\mathrm{interf}} \right)\boldsymbol{)}$

4. $\boldsymbol{B}_{1}^{+}=\frac{1}{I_{A}}\left( \mathbf{M}^{T} \right)^{-1}\boldsymbol{\alpha}_{\mathrm{interf}}$.

Although it provides a more noise-robust B_1_^+^ estimate than the HybM, the pairwise approach requires a substantially longer acquisition time and still involves signal division (in step 1), thereby remaining susceptible to noise amplification.

## **S2. Estimation of the SoS transmit and receive profiles (step 3 of FitM)**

In Step 3 of the FitM B_1_ reconstruction algorithm, the SoS transmit ($\sigma^{+}$) and receive ($\sigma^{-}$) profiles are recovered from the combined signal $\boldsymbol{d}$ by solving the joint optimization problem:

$\left( \hat{\sigma^{+}}, \hat{\sigma^{-}} \right)=\underset{\sigma^{+}, \sigma^{-}}{\mathrm{argmin}} \left( \left\| \boldsymbol{d}-\sigma^{-}\boldsymbol{s}\left( \sigma^{+}\hat{\boldsymbol{b}_{1}^{+}} \right) \right\|_{2} \right)$	[S4]

where $\boldsymbol{s}\left( \cdot\right)$ is given by Equation 2, and $\hat{\boldsymbol{b}_{1}^{+}}$ was estimated in Step 2 and is held fixed throughout the optimization. The estimate $\hat{\sigma^{+}}$ can be obtained by solving the following equivalent nested optimization problem:

$\hat{\sigma^{+}}=\underset{\sigma^{+}}{\mathrm{argmin}}\left( \min_{\sigma^{-}}\left( \left\| \boldsymbol{d}-\sigma^{-}\boldsymbol{s}\left( \sigma^{+}\hat{b_{1}^{+}} \right) \right\|_{2} \right) \right)$	[S5]

For each fixed value of $\sigma^{+}$, the inner minimization in Eq. S5 is a linear least-squares problem in $\sigma^{-}$; therefore, its solution $\tilde{\sigma}^{-}\left( \sigma^{+} \right)$ can be expressed in closed form as:

$\tilde{\sigma}^{-}\left( \sigma^{+} \right)=\frac{{\boldsymbol{s}\left( \sigma^{+}\hat{\boldsymbol{b}_{1}^{+}} \right)}^{H}\boldsymbol{d}}{{\boldsymbol{s}\left( \sigma^{+}\hat{\boldsymbol{b}_{1}^{+}} \right)}^{H}\boldsymbol{s}\left( \sigma^{+}\hat{\boldsymbol{b}_{1}^{+}} \right)}$	[S6]

Substituting Eq. S6 into Eq. S5 eliminates $\sigma^{-}$ and yields a one-dimensional nonlinear optimization problem:

$\hat{\sigma^{+}}=\underset{\sigma^{+}}{\mathrm{argmin}}\left( \left\| \boldsymbol{d}-\tilde{\sigma}^{-}\left( \sigma^{+} \right)\boldsymbol{s}\left( \sigma^{+}\hat{\boldsymbol{b}_{1}^{+}} \right) \right\|_{2} \right)$	[S7]

Finally, once $\hat{\sigma^{+}}$ has been determined, the corresponding estimate of the SoS receive profile is recovered from Eq. S6 as:

$\hat{\sigma^{-}}=\tilde{\sigma}^{-}\left( \hat{\sigma^{+}} \right)$	[S8]

As a result, the original problem (Eq. S4) is reduced to a one-dimensional non-linear least-squares problem in $\sigma^{+}$ (Eq. S7), while $\sigma^{-}$ is recovered analytically (Eq. S6). Although the resulting objective function is non-convex, it can be solved numerically at a reasonable computational cost due to the limited dynamic range of the SoS transmit profile. In practice, a standard nonlinear optimization algorithm (e.g., SQP) initialized with the median value of the *reference* $\sigma^{+}$ distribution was found to converge reliably.

## **Table S1. Notation Used**

| **Symbol** | **Description** | **Dimensions** | **Units** |
| --- | --- | --- | --- |
| $N_{\mathrm{TX}}$ | Number of transmit channels | $\mathbb{R}^{1}$ | - |
| $N_{\mathrm{RX}}$ | Number of receive channels | $\mathbb{R}^{1}$ | - |
| $N_{\mathrm{LCC}}$ | Number of applied linear channel combinations (total number of acquisitions) | $\mathbb{R}^{1}$ | - |
| $N_{\mathrm{SAT}}$ | Number of acquisitions with pre-saturation | $\mathbb{R}^{1}$ | - |
| $N_{\mathrm{NoSAT}}$ | Number of acquisitions without pre-saturation | $\mathbb{R}^{1}$ | - |
| $N_{v}$ | Number of voxels in the reference B_1_^+^ maps | $\mathbb{R}^{1}$ | - |
| $\boldsymbol{B}_{1}^{+}$ | Voxel-wise transmit sensitivities | $\mathbb{C}^{N_{\mathrm{TX}}\times1}$ | rad∙V^-1^∙s^-1^ |
| $\sigma^{+}$ | Sum-of-squares transmit profile | $\mathbb{R}^{1}$ | rad∙V^-1^∙s^-1^ |
| $\boldsymbol{b}_{1}^{+}$ | Transmit phasor | $\mathbb{C}^{N_{\mathrm{TX}}\times1}$ | dimensionless (unit-norm) |
| $\boldsymbol{B}_{1}^{-}$ | Voxel-wise receive sensitivities | $\mathbb{C}^{N_{\mathrm{RX}}\times1}$ | arbitrary units |
| $\sigma^{-}$ | Sum-of-squares receive profile | $\mathbb{R}^{1}$ | arbitrary units |
| $\boldsymbol{b}_{1}^{-}$ | Receive phasor | $\mathbb{C}^{N_{\mathrm{RX}}\times1}$ | dimensionless (unit-norm) |
| $\alpha$  $\beta$ | Voxel-wise saturation and readout flip angles | $\mathbb{R}^{1}$ | rad |
| $I_{A}$  $I_{B}$ | RF pulse integrals of the saturation and readout pulses | $\mathbb{R}^{1}$ | V∙s |
| $\boldsymbol{x}$  $\boldsymbol{y}$ | Linear channel combinations for the saturation and readout RF pulses | $\mathbb{C}^{N_{\mathrm{TX}}\times1}$ | dimensionless (unit-norm) |
| $\mathbf{X}$  $\mathbf{Y}$ | Encoding matrices regrouping the LCCs for the saturation and readout RF pulses | $\mathbb{C}^{N_{\mathrm{TX}}\times N_{\mathrm{LCC}}}$ | dimensionless |
| $\mathbf{X}^{(sat)}$  $\mathbf{Y}^{(sat)}$ | Encoding matrices containing the LCCs played in the pre-saturated acquisitions | $\mathbb{C}^{N_{\mathrm{TX}}\times N_{\mathrm{SAT}}}$ | dimensionless |
| $\mathbf{X}^{(0)}$  $\mathbf{Y}^{(0)}$ | Encoding matrices containing the LCCs used for acquisitions without pre-saturation | $\mathbb{C}^{N_{\mathrm{TX}}\times N_{\mathrm{NoSAT}}}$ | dimensionless |
| $\mathbf{S}$ | Matrix representation of the signal model across different LCCs and receive channels | $\mathbb{C}^{N_{\mathrm{LCC}}\times N_{\mathrm{RX}}}$ | arbitrary units |
| $\boldsymbol{s}(\boldsymbol{B}_{1}^{+})$ | Transmit-related components of the signal model | $\mathbb{C}^{N_{\mathrm{LCC}}\times1}$ | dimensionless |
| $\mathbf{D}$ | Signal acquired with different LCCs and across all receive channels | $\mathbb{C}^{N_{\mathrm{LCC}}\times N_{\mathrm{RX}}}$ | arbitrary units |
| $\boldsymbol{d}$ | Combined signals obtained after applying SVD to the multi-Rx signal | $\mathbb{C}^{N_{\mathrm{LCC}}\times1}$ | arbitrary units |
| $\boldsymbol{d}^{(0)}$ | Portion of the combined signals corresponding to the non-saturated acquisitions | $\mathbb{C}^{N_{\mathrm{NoSAT}}\times1}$ | arbitrary units |
| $\boldsymbol{d}^{(sat)}$ | Portion of the combined signals corresponding to the pre-saturated acquisitions | $\mathbb{C}^{N_{\mathrm{SAT}}\times1}$ | arbitrary units |
| $\mathbf{B}_{1,ref}^{+}$ | Matrix of reference transmit sensitivities across transmit channels and voxels of interest | $\mathbb{C}^{N_{\mathrm{TX}}\times N_{v}}$ | rad∙V^-1^∙s^-1^ |
| $\boldsymbol{p}_{\boldsymbol{z}}$ | Normalized flip-angle pattern of LCC $\boldsymbol{z}$ across voxels | $\mathbb{R}^{1\times N_{v}}$ | dimensionless |
| $\mathbf{P}_{\mathbf{Z}}$ | Normalized FA patterns across all voxels and $n$ LCCs defined by encoding matrix $\mathbf{Z}$ | $\mathbb{R}^{n\times N_{v}}$ | dimensionless |

## **Table S2. FitM evaluation for the 7T Avanti2 and 11.7T Iseult coils using spherical phantom simulations**

Comparison of HybM, PwM, and FitM B_1_ reconstruction methods for the 7T Avanti2 and 11.7T Iseult coils using **spherical phantom** simulations. $N_{\mathrm{NoSAT}}$ and $N_{\mathrm{SAT}}$ correspond to the number of acquisitions without and with pre-saturation, respectively. The total number of satTFL images is $N_{\mathrm{LCC}}=N_{\mathrm{NoSAT}}+N_{\mathrm{SAT}}$, acquired in TA. $\Delta_{\mathrm{TX}}$ and $\Delta_{\mathrm{RX}}$ denote the reconstruction errors for the transmit and receive sensitivities respectively, calculated from synthetic noise-added data. $\mathrm{Med}(\Delta)$ is the median error, $P_{99}(\Delta)$ is the 99% percentile and $D_{30}\left( \Delta\right)$ is the fraction of voxels with an error exceeding 30%. 〈α〉±σ(α) (resp. 〈β〉±σ(β)) is the mean±std of the FA distributions across all LCCs in the spherical phantom for the saturation (resp. readout) pulse.

|  | $\mathbf{HybM}$ | $\mathbf{PwM}$ | $\mathbf{FitM}$ | $\mathbf{FitM}$ | $\mathbf{FitM}$ | $\mathbf{FitM}$ | $\mathbf{FitM}$ | $\mathbf{FitM}$ | $\mathbf{FitM}$ | $\mathbf{FitM}$ |
| --- | --- | --- | --- | --- | --- | --- | --- | --- | --- | --- |
| $\mathbf{N}_{\mathbf{NoSAT}}$ | 9 | 9 | 8 | 8 | 8 | 8 | 8 | 8 | 8 | 8 |
| $\mathbf{N}_{\mathbf{SAT}}$ | 1 | 8 | 1 | 2 | 3 | 4 | 5 | 6 | 7 | 8 |
| **7 T Avanti2 coil** | | | | | | | | | | |
| **TA, min:sec** | 02:00 | 04:20 | 01:40 | 02:00 | 02:20 | 02:40 | 03:00 | 03:20 | 03:40 | 04:00 |
| $\left\langle\boldsymbol{\alpha} \right\rangle\boldsymbol{\pm}\boldsymbol{\sigma}\left( \boldsymbol{\alpha} \right)\boldsymbol{,^{\circ}}$ | 84.8±  23.6 | 60.3±  24.9 | 84.8±  23.6 | 66.3±  19.9 | 68.8±  24.1 | 70.7±  28.0 | 66.0±  24.6 | 68.5±  26.4 | 63.3±  24.9 | 52.8±  21.8 |
| $\left\langle\boldsymbol{\beta} \right\rangle\boldsymbol{\pm}\boldsymbol{\sigma}\left( \boldsymbol{\beta} \right)\boldsymbol{,^{\circ}}$ | 7.8±  3.7 | 9.8±  3.6 | 7.8±  3.1 | 8.4±  3.3 | 8.3±  3.3 | 8.5±  3.5 | 7.9±  3.1 | 8.7±  3.5 | 8.4±  3.4 | 7.5±  3.1 |
| $\mathbf{Med}\boldsymbol{(}\boldsymbol{\Delta}_{\mathbf{TX}}\boldsymbol{)}$**, %** | 1.26 | 1.26 | 1.25 | 1.15 | 1.12 | 1.10 | 1.06 | 1.08 | 1.06 | 1.05 |
| $\mathbf{P}_{\mathbf{99}}\boldsymbol{(}\boldsymbol{\Delta}_{\mathbf{TX}}\boldsymbol{)}$**, %** | 9.46 | 10.46 | 20.39 | 2.67 | 2.76 | 2.72 | 2.93 | 2.64 | 2.81 | 3.75 |
| $\mathbf{D}_{\mathbf{30}}\boldsymbol{(}\boldsymbol{\Delta}_{\mathbf{TX}}\boldsymbol{)}$**,%** | 0.43 | 0.06 | 0.69 | 0.00 | 0.00 | 0.00 | 0.00 | 0.00 | 0.00 | 0.00 |
| $\mathbf{Med}\boldsymbol{(}\boldsymbol{\Delta}_{\mathbf{RX}}\boldsymbol{)}$**, %** | 0.55 | 0.20 | 0.52 | 0.35 | 0.33 | 0.31 | 0.27 | 0.27 | 0.27 | 0.29 |
| $\mathbf{P}_{\mathbf{99}}\boldsymbol{(}\boldsymbol{\Delta}_{\mathbf{RX}}\boldsymbol{)}$**, %** | 9.48 | 3.64 | 25.03 | 1.65 | 1.56 | 1.41 | 1.67 | 1.52 | 1.61 | 2.40 |
| $\mathbf{D}_{\mathbf{30}}\boldsymbol{(}\boldsymbol{\Delta}_{\mathbf{RX}}\boldsymbol{)}$**,%** | 0.41 | 0.00 | 0.90 | 0.00 | 0.00 | 0.00 | 0.00 | 0.00 | 0.00 | 0.01 |
| **11.7 T Iseult coil** | | | | | | | | | | |
| **TA, min:sec** | 02:30 | 04:15 | 02:15 | 02:30 | 02:45 | 03:00 | 03:15 | 03:30 | 03:45 | 04:00 |
| $\left\langle\boldsymbol{\alpha} \right\rangle\boldsymbol{\pm}\boldsymbol{\sigma}\left( \boldsymbol{\alpha} \right)\boldsymbol{,^{\circ}}$ | 66.5±  22.5 | 45.5±  19.9 | 66.5±  22.5 | 60.9±  22.8 | 65.5±  24.7 | 61.3±  23.0 | 57.1±  23.0 | 55.2±  21.4 | 48.6±  20.2 | 45.5±  19.9 |
| $\left\langle\boldsymbol{\beta} \right\rangle\boldsymbol{\pm}\boldsymbol{\sigma}\left( \boldsymbol{\beta} \right)\boldsymbol{,^{\circ}}$ | 8.1±  3.4 | 8.1±  3.1 | 8.1±  3.5 | 8.1±  3.4 | 7.9±  3.3 | 8.1±  3.4 | 8.1±  3.4 | 7.4±  3.1 | 7.8±  3.3 | 8.1±  3.5 |
| $\mathbf{Med}\boldsymbol{(}\boldsymbol{\Delta}_{\mathbf{TX}}\boldsymbol{)}$**, %** | 1.31 | 1.62 | 1.32 | 1.20 | 1.16 | 1.13 | 1.12 | 1.09 | 1.10 | 1.11 |
| $\mathbf{P}_{\mathbf{99}}\boldsymbol{(}\boldsymbol{\Delta}_{\mathbf{TX}}\boldsymbol{)}$**, %** | 38.55 | 23.31 | 50.43 | 3.92 | 2.79 | 2.59 | 2.68 | 2.85 | 2.78 | 2.95 |
| $\mathbf{D}_{\mathbf{30}}\boldsymbol{(}\boldsymbol{\Delta}_{\mathbf{TX}}\boldsymbol{)}$**, %** | 1.23 | 0.53 | 1.66 | 0.02 | 0.00 | 0.00 | 0.00 | 0.00 | 0.00 | 0.00 |
| $\mathbf{Med}\boldsymbol{(}\boldsymbol{\Delta}_{\mathbf{RX}}\boldsymbol{)}$**, %** | 0.59 | 0.46 | 0.58 | 0.41 | 0.36 | 0.30 | 0.30 | 0.27 | 0.28 | 0.35 |
| $\mathbf{P}_{\mathbf{99}}\boldsymbol{(}\boldsymbol{\Delta}_{\mathbf{RX}}\boldsymbol{)}$**, %** | 37.06 | 10.87 | 96.50 | 3.56 | 1.97 | 1.68 | 1.73 | 1.67 | 1.72 | 2.25 |
| $\mathbf{D}_{\mathbf{30}}\boldsymbol{(}\boldsymbol{\Delta}_{\mathbf{RX}}\boldsymbol{)}$**, %** | 1.21 | 0.10 | 1.93 | 0.03 | 0.00 | 0.00 | 0.00 | 0.00 | 0.00 | 0.00 |

## **Table S3. FitM evaluation for the 7T Avanti2 and 11.7T Iseult coils using head model simulations**

Evaluation of FitM for the 7T Avanti2 and 11.7T Iseult coils using **head model** simulations and the phantom-derived acquisition schemes with N_SAT_=1…8. $\Delta_{\mathrm{TX}}$ and $\Delta_{\mathrm{RX}}$ denote the reconstruction errors for the transmit and receive sensitivities respectively, calculated from synthetic noise-added data. $\mathrm{Med}(\Delta)$ is the median error, $P_{99}(\Delta)$ is the 99% percentile and $D_{30}\left( \Delta\right)$ is the fraction of voxels with an error exceeding 30%. 〈α〉±σ(α) (resp. 〈β〉±σ(β)) is the mean±std of the FA distributions across all LCCs in the spherical phantom for the saturation (resp. readout) pulse.

|  | $\mathbf{FitM}$  $\mathbf{N}_{\mathbf{SAT}}\boldsymbol{=1}$ | $\mathbf{FitM}$  $\mathbf{N}_{\mathbf{SAT}}\boldsymbol{=2}$ | $\mathbf{FitM}$  $\mathbf{N}_{\mathbf{SAT}}\boldsymbol{=3}$ | $\mathbf{FitM}$  $\mathbf{N}_{\mathbf{SAT}}\boldsymbol{=4}$ | $\mathbf{FitM}$  $\mathbf{N}_{\mathbf{SAT}}\boldsymbol{=5}$ | $\mathbf{FitM}$  $\mathbf{N}_{\mathbf{SAT}}\boldsymbol{=6}$ | $\mathbf{FitM}$  $\mathbf{N}_{\mathbf{SAT}}\boldsymbol{=7}$ | $\mathbf{FitM}$  $\mathbf{N}_{\mathbf{SAT}}\boldsymbol{=8}$ |
| --- | --- | --- | --- | --- | --- | --- | --- | --- |
| **7 T Avanti2 coil** | | | | | | | | |
| $\left\langle\boldsymbol{\alpha} \right\rangle\boldsymbol{\pm}\boldsymbol{\sigma}\left( \boldsymbol{\alpha} \right)\boldsymbol{,^{\circ}}$ | 74.0±  27.9 | 62.6±  26.6 | 63.7±  27.4 | 63.2±  28.2 | 56.5±  25.0 | 59.7±  23.8 | 55.6±  23.9 | 44.9±  19.8 |
| $\left\langle\boldsymbol{\beta} \right\rangle\boldsymbol{\pm}\boldsymbol{\sigma}\left( \boldsymbol{\beta} \right)\boldsymbol{,^{\circ}}$ | 6.7±  2.9 | 7.3±  3.2 | 7.2±  3.2 | 7.3±  3.3 | 6.8±  3.0 | 7.5±  3.2 | 7.3±  3.2 | 6.4±  2.8 |
| $\mathbf{Med}\boldsymbol{(}\boldsymbol{\Delta}_{\mathbf{TX}}\boldsymbol{)}$**, %** | 1.34 | 1.16 | 1.13 | 1.09 | 1.10 | 1.06 | 1.06 | 1.12 |
| $\mathbf{P}_{\mathbf{99}}\boldsymbol{(}\boldsymbol{\Delta}_{\mathbf{TX}}\boldsymbol{)}$**, %** | 48.96 | 8.54 | 5.68 | 2.56 | 2.49 | 2.36 | 2.37 | 2.75 |
| $\mathbf{D}_{\mathbf{30}}\boldsymbol{(}\boldsymbol{\Delta}_{\mathbf{TX}}\boldsymbol{)}$**, %** | 2.08 | 0.21 | 0.01 | 0.00 | 0.00 | 0.00 | 0.00 | 0.00 |
| $\mathbf{Med}\boldsymbol{(}\boldsymbol{\Delta}_{\mathbf{RX}}\boldsymbol{)}$**, %** | 0.60 | 0.40 | 0.34 | 0.29 | 0.27 | 0.25 | 0.25 | 0.29 |
| $\mathbf{P}_{\mathbf{99}}\boldsymbol{(}\boldsymbol{\Delta}_{\mathbf{RX}}\boldsymbol{)}$**, %** | 93.64 | 8.72 | 5.86 | 1.61 | 1.44 | 1.27 | 1.31 | 1.60 |
| $\mathbf{D}_{\mathbf{30}}\boldsymbol{(}\boldsymbol{\Delta}_{\mathbf{RX}}\boldsymbol{)}$**, %** | 2.35 | 0.24 | 0.15 | 0.04 | 0.00 | 0.01 | 0.00 | 0.00 |
| **11.7 T Iseult coil** | | | | | | | | |
| $\left\langle\boldsymbol{\alpha} \right\rangle\boldsymbol{\pm}\boldsymbol{\sigma}\left( \boldsymbol{\alpha} \right)\boldsymbol{,^{\circ}}$ | 55.8±  23.6 | 48.6±  21.1 | 51.7±  23.6 | 49.3±  22.5 | 45.7±  21.6 | 44.4±  20.3 | 38.3±  19.4 | 36.1±  17.8 |
| $\left\langle\boldsymbol{\beta} \right\rangle\boldsymbol{\pm}\boldsymbol{\sigma}\left( \boldsymbol{\beta} \right)\boldsymbol{,^{\circ}}$ | 6.5±  3.1 | 6.4±  3.1 | 6.2±  3.0 | 6.4±  3.1 | 6.4±  3.1 | 5.9±  2.8 | 6.2±  3.1 | 6.4±  3.2 |
| $\mathbf{Med}\boldsymbol{(}\boldsymbol{\Delta}_{\mathbf{TX}}\boldsymbol{)}$**, %** | 1.39 | 1.28 | 1.22 | 1.17 | 1.16 | 1.20 | 1.17 | 1.16 |
| $\mathbf{P}_{\mathbf{99}}\boldsymbol{(}\boldsymbol{\Delta}_{\mathbf{TX}}\boldsymbol{)}$**, %** | 97.69 | 30.00 | 6.85 | 5.01 | 5.26 | 3.66 | 4.00 | 4.29 |
| $\mathbf{D}_{\mathbf{30}}\boldsymbol{(}\boldsymbol{\Delta}_{\mathbf{TX}}\boldsymbol{)}$**, %** | 4.22 | 1.00 | 0.10 | 0.02 | 0.01 | 0.00 | 0.00 | 0.00 |
| $\mathbf{Med}\boldsymbol{(}\boldsymbol{\Delta}_{\mathbf{RX}}\boldsymbol{)}$**, %** | 0.70 | 0.52 | 0.41 | 0.34 | 0.34 | 0.32 | 0.33 | 0.38 |
| $\mathbf{P}_{\mathbf{99}}\boldsymbol{(}\boldsymbol{\Delta}_{\mathbf{RX}}\boldsymbol{)}$**, %** | 1595.70 | 35.42 | 6.55 | 4.54 | 4.75 | 2.80 | 3.27 | 3.57 |
| $\mathbf{D}_{\mathbf{30}}\boldsymbol{(}\boldsymbol{\Delta}_{\mathbf{RX}}\boldsymbol{)}$**, %** | 4.61 | 1.12 | 0.11 | 0.03 | 0.02 | 0.00 | 0.00 | 0.00 |

## **Figure S1. In vivo B_1_^-^ maps, 7T Avanti2 coil**

7T in vivo data acquired in six participants with the Avanti2 coil using N_SAT_=3 acquisition scheme (TA: 2 min 30 s) and reconstructed with the FitM approach. Magnitude (A) and phase (B) of the in vivo receive sensitivities B_1_^-^, modulated by proton density, for each of the 32 Avanti2 receive channels.
